# Trial Refresh: A Case for an Adaptive Platform Trial for Pulmonary Exacerbations of Cystic Fibrosis

**DOI:** 10.3389/fphar.2019.00301

**Published:** 2019-03-28

**Authors:** Andre Schultz, Julie A. Marsh, Benjamin R. Saville, Richard Norman, Peter G. Middleton, Hugh W. Greville, Matthew I. Bellgard, Scott M. Berry, Tom Snelling

**Affiliations:** ^1^Faculty of Health and Medical Sciences, The University of Western Australia, Crawley, WA, Australia; ^2^Department of Respiratory Medicine, Perth Children’s Hospital, Nedlands, WA, Australia; ^3^Wesfarmers Centre of Vaccines & Infectious Diseases, Telethon Kids Institute, The University of Western Australia, Nedlands, WA, Australia; ^4^School of Population and Global Health, The University of Western Australia, Nedlands, WA, Australia; ^5^Berry Consultants, Austin, TX, United States; ^6^Department of Biostatistics, Vanderbilt University, Nashville, TN, United States; ^7^School of Public Health, Curtin University, Bentley, WA, Australia; ^8^Ludwig Engel Centre for Respiratory Research, Westmead Institute for Medical Research, Sydney, NSW, Australia; ^9^Department of Thoracic Medicine, Royal Adelaide Hospital, Adelaide, SA, Australia; ^10^eResearch Office, Queensland University of Technology, Brisbane, QLD, Australia; ^11^Department of Infectious Diseases, Perth Children’s Hospital, Nedlands, WA, Australia; ^12^Menzies School of Health Research, Charles Darwin University, Darwin, NT, Australia

**Keywords:** adaptive trial, platform trial, Bayesian, cystic fibrosis, exacerbations, master protocol, response adaptive randomisation

## Abstract

Cystic fibrosis is a genetic disease typically characterized by progressive lung damage and premature mortality. Pulmonary exacerbations, or flare-ups of the lung disease, often require hospitalization for intensive treatment. Approximately 25% of patients with cystic fibrosis do not recover their baseline lung function after pulmonary exacerbations. There is a relative paucity of evidence to inform treatment strategies for exacerbations. Compounding this lack of evidence, there are a large number of treatment options already as well as becoming available. This results in significant variability between medication regimens prescribed by different physicians, treatment centers and regions with potentially adverse impact to patients. The conventional strategy is to undertake essential randomized clinical trials to inform treatment decisions and improve outcomes for patients with exacerbations. However, over the past several decades, clinical trials have generally failed to provide information critical to improved treatment and management of exacerbations. Bayesian adaptive platform trials hold the promise of addressing clinical uncertainties and informing treatment. Using modeling and response adaptive randomization, they allow for the evaluation of multiple treatments across different management domains, and progressive improvement in patient outcomes throughout the course of the trial. Bayesian adaptive platform trials require substantial amounts of preparation. Basic preparation includes extensive stakeholder involvement including elicitation of consumer preferences and clinician understanding of the research topic, defining the research questions, determining the best outcome measures, delineating study sub-groups, in depth statistical modeling, designing end-to-end digital solutions seamlessly supporting clinicians, researchers and patients, constructing randomisation algorithms and importantly, defining pre-determined intra-study end-points. This review will discuss the motivation and necessary steps required to embark on a Bayesian adaptive platform trial to optimize medication regimens for the treatment of pulmonary exacerbations of cystic fibrosis.

## Background

Cystic fibrosis (CF) is a genetic disease typically characterized by progressive lung damage and premature mortality. Pulmonary exacerbations, or flare-ups of the lung disease, often require hospitalization for intensive treatment. Pulmonary exacerbations remain an important driver of progressive loss of lung function and premature death in CF. Up to 25% of patients do not recover their baseline lung function, typically measured as the forced expiratory volume in 1 s (FEV1), after an exacerbation (Sanders et al., 2010). Key to improving survival is the prevention of loss of lung function with each exacerbation. Reflecting this, when the James Lind Alliance recently canvassed research priorities from over 1,000 CF consumers from 23 countries, management of CF exacerbations, and specifically identification of the most effective/least toxic antibiotics, figured among their priorities (Rowbotham et al., 2018).

There are grounds for believing that better treatment of exacerbations may reduce the loss of lung function and thus improve survival. There is substantial variation in the rate of decline between individuals, even among those with the same primary genetic mutations. The demonstrated heterogeneity in outcomes among those with access to the same range of treatments (Stephenson et al., 2017), indicates that response may depend on individual patient characteristics or factors present during an exacerbation. Given these considerations, we identify three general questions that need to be addressed in order to improve the management of CF exacerbations: (a) What are the optimal interventions where multiple options exist; (b) How does optimal treatment vary by different patient characteristics; and (c) Do treatments have cumulative or antagonistic effects when used in combination.

Despite considerable research effort, answers to these questions have remained elusive. Antibiotics are a mainstay of the treatment of pulmonary exacerbations. A recent Cochrane review identified 40 studies of antibiotic treatment of CF exacerbations (Hurley et al., 2015). Most studies evaluated only two antibiotics and were small, inconclusive, never replicated, and completed a decade or more ago. The reviewers concluded that “No specific antibiotic combination can be considered to be superior to any other, and neither is there evidence showing that the intravenous route is superior to the inhaled or oral routes” (Hurley et al., 2015). The failure of this review of several decades of research to reach any meaningful conclusion is symptomatic of the challenges of conducting trials in CF. Continuing to undertake conventional trials may never usefully address this clinical uncertainty, and certainly not within a timeframe that will benefit many of the current generation of people with CF.

Recent advances in clinical trial methods can help to evaluate complex interventions for rare conditions like CF. These innovations allow for improved trial efficiency and may be more conducive to participation by patients and clinicians alike. These methods have recognized validity, having been used particularly in commercial cancer trials, accepted for regulatory purposes by both the Food and Drug Administration (Woodcock and LaVange, 2017) and the European Medicines Agency, and published in high impact journals. These methods are being applied in trials of the treatment of breast cancer (I-SPY2) (Park et al., 2016), severe community-acquired pneumonia (REMAP-CAP (NCT02735707)), and brain cancer (GBM-AGILE) (Alexander et al., 2018). Here, we describe the planning for BEAT CF, a Bayesian adaptive platform trial that aims to optimize the management of CF exacerbations.

BEAT CF aims to be an exemplar of the REMAP (randomized, embedded, multi-arm, adaptive, platform) trial approach (Angus, 2015). The key features of BEAT CF are the use of Bayesian statistical inference, flexible sample size, the comparison of several different treatment options simultaneously and in combination, response adaptive randomization, and the evaluation of treatment responses in different types of patient. Key to the successful implementation of BEAT CF will be its nesting within a treatment register, embedding of trial procedures in routine clinical care and a digital health approach that is dynamic to adapt to the informatics and data integration challenges (Bellgard et al., 2017). A core (master) protocol will allow the sequential introduction of new interventions over time, as initial questions are answered. Each of these features will be introduced below, contrasting REMAP trials with more traditional trial approaches, and highlighting the benefits, challenges and limitations inherent in establishing a REMAP trial for a complex clinical domain.

## Core Protocol

A limitation of undertaking sequential disconnected trials is that the lack of standardized eligibility criteria, trial endpoints, subgroup definitions, and comparator treatments makes the aggregation of such data complex and uncertain. The core protocol of a REMAP trial aims to standardize these design elements so that treatment responses can be meaningfully aggregated across trials, across settings and over time. For BEAT CF, we propose to broadly involve clinicians and other domain experts, consumers and other stakeholders in this decision-making. Investment in the development of an overarching core protocol is also intended to prevent *reinventing the wheel* for each trial, improving efficiency through the sharing of infrastructure, and reducing the time to commencement and completion. In addition to standardizing specific design elements, the core protocol can also set out an overarching governance framework, including how the safety of participants is monitored. A core protocol implemented across multiple centers might institute a platform for the ready identification of potentially eligible study participants, facilitating enrolments.

## Bayesian Statistical Inference

Up until now, most clinical trials, and all trials in managing CF exacerbations, have been traditionally designed trials which have employed frequentist statistical inference. Frequentist statistical inference underlies the vast majority of clinical studies, although thanks to increasing computer processing capacity, there has been a steady resurgence in Bayesian statistical inference in recent years (Green et al., 2015). In brief, frequentist inference assesses the *likelihood* of an observation, such as the observed difference in treatment effect between an investigational and a control treatment if no true difference exists (Berry, 2006). If an observation is very unlikely under this “null hypothesis” (i.e., it would rarely occur just by chance), this is taken as evidence that, to the contrary, a true difference exists. Frequentist inference flips the question of the probability that a difference exists, into a question about the probability of a result if no difference exists. To apply frequentist inference, one needs to be able to enumerate all the possible ways that a trial could have unfolded (that is the possible number of treatment successes and failures across both arms), and this requires many aspects of the design to be fixed in advance (e.g., randomization probabilities, sample sizes, number of treatment arms). If these components are not fixed it may be impossible to enumerate the number of possible ways that a trial may have unfolded and therefore to calculate how “unlikely” an observation is. Fixing the design so as to confine the number of possible outcomes makes the frequentist analysis tractable, but it comes at the cost of lost flexibility. New methods allow the design to be adapted as data accrues according to established rules (Saville et al., 2014), and these rules can be designed to maximize efficiency (including the chance of a conclusive result), or to maximize the chance that participants receive optimal treatment, or both.

Whereas frequentist inference is based solely on the *likelihood of an outcome*, Bayesian inference is directly concerned with the actual question, i.e., the *probability of a difference in treatment effect*, or the most probable values of the true difference in treatment effects. Bayesian inference does this by combining the *likelihood* of the observation for the range of possible treatment differences with the baseline *probability* (or “prior”) of those possible treatment differences (Dmitrienko and Wang, 2006). Bayesian inference provides a straight-forward mechanism for updating one’s estimate of the most probable range of treatment differences as new observations are made, that is, as data accrues (Connor et al., 2013). Trial designs that are adaptive can unfold in any one of a nearly limitless number of ways, so estimating how “unlikely” a particular result is (that is a particular instance of treatment responses among those who receive an intervention or control) is very hard to determine. The ability to update the probabilities for a range of possible treatment differences as new data accrues therefore makes Bayesian inference very useful for adaptive studies, although it should be noted that adaptive designs based on frequentist inference have also been advocated.

## Response Adaptive Randomisation

When we have surveyed Australian CF clinicians, we have found that, like colleagues in the United States (West et al., 2017), they use a wide range of antibiotics to treat pulmonary exacerbations. Sequentially comparing the relative efficacy of all currently used antibiotics two-at-a-time would take an unfeasibly long time, notwithstanding the complexity of antibiotic combinations. Furthermore, assessing all antibiotics contemporaneously in a multi-arm trial would require an unfeasibly large number of participants. REMAP trials aim to greatly improve the efficiency of multi-arm trials using response adaptive randomisation (RAR). RAR is the progressive, rule-based assignment of an increasing proportion of new participants to interventions which appear most promising, and the potential elimination of those which are demonstrated to be inferior to alternatives options (Berry et al., 2015; Cellamare et al., 2017) (Box 1). Rather than having a fixed ratio of treatment assignment across arms, the ratio is updated (or adapted) at predefined intervals based on evidence from accruing data (Connor et al., 2013). At each analysis we will estimate the probability that each treatment, or treatment combination, is superior to all other options for a given patient type. Future treatment assignments will be based on these probabilities such that probability of assignment to a treatment is proportional to the probability that treatment is better than all other options for a given patient type (Connor et al., 2013). Randomization to ineffective treatments may be suspended or entirely eliminated if pre-specified futility boundaries are met (Berry et al., 2015). By assigning progressively more randomized participants to the best strategies and dropping ineffective therapies, it is expected that RAR will produce better patient outcomes for patients who participate in the trial (Connor et al., 2013).

**Box 1. Response adaptive randomisation.**

• The progressive, rule-based assignment of an increasing proportion of new participants to interventions which appear most promising.
• The potential elimination of interventions demonstrated to be inferior to available alternatives when pre-specified futility boundaries are met.

## Subgroup Effects

One challenge for clinicians when trying to apply clinical trial results to their clinical practice is in determining whether the results are applicable to their own patient population, or indeed to a specific patient at hand. In pulmonary exacerbations, for example, it seems highly plausible that exacerbations represent a range of pathophysiologic processes, with responses to certain treatments influenced by the predominant process and, in the case of exacerbations caused by infecting bacteria, the species, and susceptibility profile of those bacteria (Doring et al., 2012). However, most trials are only interested in measuring *average* treatment effects of an intervention over a study population, and implicitly assume individuals respond homogenously to different interventions or treatment combinations. If a difference in treatment effects between subgroups is expected, the trialist will need to confine the trial to only the patient group which is expected to respond, or will run parallel trials in each group. If a difference in treatment response is hypothesized but not expected, the trialist can choose to aggregate data across the subgroups unless a statistical test for heterogeneity confirms a difference in treatment effects across subgroups, in which case the results for the two groups are reported separately. The problem with the latter approach is that such tests are relatively insensitive for true differences which can lead to inappropriate pooling of results; the problem with splitting by patient subgroups is that the precision is diluted by ever shrinking sample sizes. This is a problem in CF where the range of potential subgroups and plausible treatment effect modifiers is very large, based on variation in sputum microbiology, disease stage, and prior and concurrent treatment history.

REMAP trials aim to estimate specific treatment responses across a range of predefined patient subgroups much more efficiently than traditional trials. Such subgroups could be defined by factors that plausibly impact on response to treatment, or more specifically, influence the probability of a better response to one treatment than another, for example baseline lung function and airway microbiology status in the case of pulmonary exacerbations. Bayesian modeling can be used to allow the observed treatment response in one group to inform the probable treatment effect in another. Bayesian prior distributions are carefully calibrated to control the amount of *borrowing* of information between respective sub groups, and are verified in the design stage via simulation (Berry and Berry, 2004). This differs from the extreme approaches of complete pooling (i.e., ignoring subgroups effects and estimating a single common treatment effect) or no pooling (i.e., independent estimation of treatment effects in each subgroup). The Bayesian modeling approach can be described as being in between these two extremes, with *partial pooling* of information from patient subgroups. For subgroups with small sample sizes, the estimated subgroup treatment effects tend to be closer to each other. As sample sizes increase within subgroups, estimation of treatment effects may grow apart depending on the observed data within the subgroups.

## Comparison of Multiple Different Treatment Options Simultaneously

Like most chronic diseases, the management of CF pulmonary exacerbations is complex and multimodal (Elborn, 2016; Waters et al., 2016; West et al., 2017). Traditional trial designs typically do not account for multimodality but instead focus on one or more treatments within a single therapeutic “domain,” where domain refers to a set of mutually exclusive treatment options. In the management of pulmonary exacerbations for example, therapeutic domains include the choice and route of antibiotic (both primary/backbone and adjunctive) (West et al., 2017), the use of mucoactive therapies (including dornase alpha, hypertonic saline, and mannitol) (Bakker et al., 2014; Dentice et al., 2016), the use of immune modulators (steroids, non-steroidal anti-inflammatory drugs, and macrolides) (Ghdifan et al., 2010; Lands and Stanojevic, 2016), and type and intensity of airway clearance therapies. Trials that attempt to evaluate a complex multi-modal intervention typically evaluate them as a fixed “bundle”; the limitation of this approach is that it makes it impossible to know which components of the bundle, if any, are effective, which ineffective or deleterious, and whether any combinations are synergistic or antagonistic.

In the REMAP design, participants can be randomly assigned to one of two or more options across each of two or more therapeutic domains. Participants are therefore assigned to one of many possible treatment combinations. This gives rise to a multi-dimensional estimation problem. For example, in a REMAP with three therapeutic domains each with three treatment options, there are 3^3^ (twenty seven) combinations and therefore 27 separate treatment effects to be estimated, ignoring any subgroup-specific effects. As for subgroup effects, this multi-dimensional estimation problem is made tractable by the use of Bayesian modeling which allows, for example, the observed treatment outcomes among patients receiving a backbone antibiotic together with one adjunctive antibiotic, to inform the estimated treatment effect of the same backbone antibiotic when given in conjunction with a different adjunctive antibiotic, and *vice versa* (Berry and Berry, 2004).

## A Platform Trial With a Decision-Based Master Protocol

Most clinical trials are stand-alone, time-limited, and designed to answer a single efficacy or comparative efficacy question. Regardless of whether the trial is conclusive or not, any follow-up or completely new questions usually require the establishment of a new trial. Typically there is little or no transfer of study infrastructure between trials which is wasteful of resources. Also, trials of interventions for CF exacerbations have variously measured different but related outcome measures, such as the absolute or relative improvements in the predicted FEV1, or the proportion of patients returning to some fraction of their “baseline” FEV1, or the reduction in some composite measure of symptoms. Furthermore, trials have measured these endpoints at variable lengths of time after initiation of CF exacerbation therapy. Failure of concurrent and consecutive trials to adopt the same endpoints has made it impossible to compare, let alone aggregate, results across studies. More recently there have been efforts to establish consistent endpoints, or core outcome sets, for trials in cystic fibrosis and other trials (Stanojevic and Ratjen, 2016; West et al., 2017).

A REMAP trial aims to achieve greater efficiency through a core (or master) trial protocol. The core protocol sets out exactly what data are to be collected including the primary and any secondary endpoints, and the procedures around how data is captured and managed, including the trial governance arrangements. Any treatment options which are subject to random assignment are dealt with in a series of appendices to the master protocol, with a therapeutic domain and all treatment options within that domain covered by its own appendix.

This use of a core protocol facilitates consistency in trial endpoints and processes over time. The modular structure of the protocol allows for the domain-specific trial appendices to be modified over time without changing the core protocol; treatment options within a domain can be added or removed according to pre-specified rules and entire domains can be added or removed over time. Once the superiority of a treatment has been established over all other treatments within an existing domain, a new domain with unanswered questions could replace the existing domain (Berry et al., 2015). This circumvents the need for setting up an entirely new clinical trial. For example if the optimal combinations of backbone and adjuvant antibiotics for CF exacerbation have been determined for individual patient sub-groups, a new phase of the study might focus on optimization of immune modifiers, mucoactive agents, airway clearance strategies or other therapeutic domains.

Non-inferiority and equivalence findings can also be evaluated in a REMAP trial. In particular, the study would still result in improvement in care if it was able to eliminate one or more inferior treatment arms, while showing the remaining treatment arms are equivalent. In addition, some treatments may require an efficacy superiority margin to be favored relative to other treatments in the presence of differing toxicity/safety profiles. We are currently investigating various options for incorporating toxicity into the primary analysis and RAR algorithm.

## Embedding in Routine Clinical Care

Despite the potential efficiencies gained through the use of adaptive processes, a REMAP trial must nonetheless enroll a large number and broad range of participants if it is to efficiently address the full range of management questions. For this to occur, a REMAP trial needs to be successfully embedded in routine clinical care. Embedding, in which trial participation occurs seamlessly with delivery of care and with minimal additional impost on either clinician or participant, requires extensive stakeholder involvement in the design and strong buy-in by clinicians and patients. To secure this, REMAP investigators must spend considerable effort eliciting clinician’s expert understanding of the subject domain, as well as consumer input into identification of patient-centered study outcomes. Clinicians and patients may be more likely to engage with a trial for which there is prospect of a personal benefit from participation. Unlike traditional trials which are ethically predicated on no expectation of a personal benefit, response adaptive processes of REMAP trials are designed to improve the chances that a participant receives optimal therapy, and minimize the chance of inferior therapy.

Many traditional trials blind the participant, or the outcome assessor, or both, to the treatment assignment, usually through the use of matched placebos or sham treatments. Blinding helps to safeguard against bias that might arise, especially if the clinician, patient or assessor have preconceptions about the relative effectiveness of the treatment options. Complete blinding of participants is typically not feasible in a REMAP owing to the large number and range of therapeutic options evaluated, together with the desire to achieve successful embedding in routine care. In any unblinded study (regardless of equal or response adaptive randomization), there is the risk that patient’s or physician’s knowledge can affect outcomes. This can be minimized through the choice of objective outcomes such as change in FEV1, especially if the person performing the measurement is, herself, blinded to the treatment assignment. For unblinded studies incorporating RAR, there is additional risk of operational bias resulting from a site’s perceived impression of adaptive randomization. Although it would be difficult to measure any such bias that might exist in this setting, we believe that knowledge of the best performing treatments at the site level would only be obvious in settings where there is a dominant treatment receiving greater randomizations across all or most subgroups. In such a setting, the risk of bias may be outweighed by the risk of continued randomization to under-performing arms.

## Digital Health Solution Considerations

The implementation of REMAP trials demands a rethink on approaches to data management, flow, sharing and the interfaces necessary to engage with participants, clinicians, and study coordinators. Whereas in conventional trials, paper-based records might be sufficient to manage the workflow and capture trial data, in a REMAP the need for continuous and iterative interaction between clinicians, patients, and statisticians demand digital solutions.

Any solution needs to ensure that data are captured electronically in order to support frequent pre-planned, scheduled analyses. Logic checks need to be built-in and designed to minimize data entry errors and the need for corrections which would otherwise delay analyses. All data needs to be held securely and privacy ensured – potentially identifiable information needs to be held at the site and not accessible to those who are not directly involved in patient care, including researchers. There should be facility for patients (or their parents) to directly enter symptom information, or patient reported outcomes (Napier et al., 2017). It is necessary for the randomisation procedure to accommodate the RAR process – that is, it is essential that any embedded randomisation process must be capable of being updated over time following each analysis. Static randomisation processes, for example those employing fixed randomisation lists, will not be fit-for-purpose.

Lessons learnt from the successful deployment of clinical registries offer insights into the types digital infrastructure required to implement REMAP trials (Bellgard et al., 2013; Lacaze et al., 2017). For instance, patient-centric registries allow patients to securely register through online registration with configurable online informed consent (Bellgard et al., 2012; Napier et al., 2017). Others have demonstrated the potential value of rich and well-collected patient registry data for improving patient decision-making in CF beyond simple rule-based algorithms (Alaa and van der Schaar, 2018). Online clinical reporting forms and participant questionnaires that can be configured by coordinators without software development skills enables the digital health solution to dynamically adapt to requirements (Bellgard et al., 2014). Longitudinal data capture and time-stamped ongoing patient assessments can be captured either by the patient themselves or by the clinician through automated notifications (Bellgard et al., 2015). In addition, patient registry platforms can have multi-lingual support and data elements can be derived allowing logic steps to be incorporated (Napier et al., 2017).

## Consent

In traditional two-arm trials, once informed consent for participation has been obtained, recording that consent is relatively straight-forward. For a REMAP trial, where a participant may be offered randomisation across a number of therapeutic domains, capturing participant consent is more complex. For example participants might happily accept random assignment of treatment in one domain, but not in another. In some situations, specific treatment options, and possibly entire domains, may not be available in all centers. In the case of a REMAP in managing CF exacerbations, a participant might decline randomisation during one exacerbation, but accept randomisation during a subsequent exacerbation. Furthermore, a participant might never accept random assignment of treatment, but might nonetheless agree to have their treatment and outcome data collected in a treatment register. Consenting to a REMAP is not simply a binary choice to participate or not, and this complex and nuanced consent needs to be captured and efficiently and faithfully reflected in subsequent study processes.

Because of the complex nature of REMAP trial designs, it may not be possible to achieve adequately informed consent at the time of acute illness. It may be necessary to provide detailed education to prospective participants in the non-acute setting. In the case of a REMAP for managing CF exacerbations, for example, an option would be to obtain consent in a dynamic and stepwise fashion. In the first step, prospective participants may be invited to consent to enrolment in a prospective treatment register, in which patients simply agree to allow their treatment and outcome data to be captured to inform future best practice. This could occur in the outpatient setting, and could be supported by extensive education about the REMAP design and processes. Separately, those who are enrolled in the treatment register can then be invited to opt-in to randomized care at the time of pulmonary exacerbations. Because participants will have already received education in the outpatient setting, this additional consent to randomisation can be expedited so as not to unnecessarily disrupt the delivery of care. In this two-level consent process, participants may agree to have their treatment and outcome data captured in the treatment register, but not always (or perhaps never) further consent to receive randomized care of their exacerbations. Similarly, the treating clinician may decide against random treatment assignment for a patient during a given exacerbation. Having patients opting out of randomisation, or their clinicians deciding against random treatment assignment, is an issue found in nearly every randomized clinical trial. Patients who opt out of randomization are not included in the primary analysis. Hence, the generalizability of the primary analysis results is limited to a population of patients who are willing to be randomized. Whereas traditional clinical trials disregard data arising from unrandomized patients as inherently uninformative due to potential bias, it may be possible within the context of a REMAP to utilize this data for hypothesis generation, or possibly even for formal integration into treatment effect estimates.

## Ethics Statement

Similar to all clinical trials BEAT CF will require approval by participating institutions’ ethics and governance committees. Such committees will be confronted with a range of innovative features including the novel study design, complex Bayesian statistics, the absence of blinding to allocated treatment, and dynamic consent. Involvement of ethics and governance committees from the early planning stages may help to avoid roadblocks in the approval process. Evidence of extensive consumer involvement in study design and planning may also be looked upon favorably by these committees.

## Trial Efficiency and Cost

REMAP trials have the potential to be highly cost-efficient as multiple research questions can be addressed simultaneously in a single clinical trial (Berry et al., 2015). Thus, while there are significant initial costs associated with establishing the trial, these costs can be defrayed across many questions of interest. Additionally, the trial platform allows for efficiency with a single data capture and governance platform across multiple centers. If REMAPs are effectively embedded in clinical care the incremental costs of inclusion of additional study sites may be relatively small, and offset by improved effectiveness and cost-efficiency of care. The use of a core protocol allows new domains to be studied without the need to develop an entirely new protocol. While REMAP designs are motivated by the desire to greatly improve the efficiency of clinical comparative effectiveness research, to date these efficiencies remain largely unproven and may only be achieved if a REMAP design aligns with the specific objectives of the study.

## Limitations and Challenges

Whilst a Bayesian adaptive platform trial many potentially provide many benefits for the study of CF exacerbations, specific challenges still remain. Some of these challenges transcend statistical design. For example, the precise definition of an exacerbation remains controversial (Goss and Burns, 2007; Stenbit and Flume, 2011). The clinical decision to hospitalize for treatment of an exacerbation may vary from one treatment center to another (Johnson et al., 2003) but has been successfully used as a pragmatic definition in clinical trials. Furthermore, the decision to admit to hospital can potentially be standardized between participating study sites (Ferkol et al., 2006). Secondly, exacerbations may have multiple etiologies requiring different optimal treatments (Stenbit and Flume, 2011). However, similar to asthma, different etiologies that result in the same clinical phenomenon (i.e., exacerbation) can usefully be studied as a single entity. Thirdly, the optimal duration of treatment for exacerbations remain contentious (Szentpetery and Flume, 2018) and there is uncertainty about best outcome measure, and timing of the outcome measure, for studying treatment effect. Such challenges face all clinical trials of exacerbations but can be overcome in the CF community where collaborative effort is the norm.

Another challenge for platform trial is population drift that can influence results. This can be addressed with Bayesian modeling that accounts for changes in population over time. Logistical challenges include the need for rapid data accrual to inform adaptations. Digital solutions, discussed above, can be applied to facilitate timely data capture. Many of the abovementioned limitations are discussed elsewhere (Saville and Berry, 2016), alongside the statistical efficiencies obtained through adaptive platform strategies.

## In Summary

There is a need to optimize the management of pulmonary exacerbations of CF, but the traditional clinical trial approach may not be a feasible approach for addressing the multitude of clinical questions. REMAP trial designs may offer a much more effective and efficient approach to finding answers to the many questions confronting CF patients and clinicians. Features include response adaptive randomisation, and the ability to compare multiple different treatment options simultaneously over a range of patient sub-groups. Once the original research questions have been answered, the platform design with a core protocol would facilitate the seamless transition to follow-up questions. For such a trial to be successful for the study of exacerbations of CF the trial will need to be embedded in routine clinical care and innovative digital solutions will be required for implementation. Overall, the challenges are large but the gains for CF could be considerable.

## Author Contributions

AS co-wrote the first draft of the manuscript and contributed to the writing throughout. JM contributed to writing the first draft. BS and MB wrote sub-sections of the manuscript and contributed the manuscript as a whole. RN, HG, and PM provided expert input into an advanced version of the manuscript. SB provided expert input and oversight. TS acted as senior author by writing large sections of the manuscript and providing oversight and leadership throughout.

## Conflict of Interest Statement

The authors declare that the research was conducted in the absence of any commercial or financial relationships that could be construed as a potential conflict of interest.
